# Bisphenol A deteriorates egg quality through HDAC7 suppression

**DOI:** 10.18632/oncotarget.21308

**Published:** 2017-09-28

**Authors:** Bin Liu, Shasha Zhou, Chenmin Yang, Ping Chen, Pingping Chen, Di Xi, Hong Zhu, Yuping Gao

**Affiliations:** ^1^ Department of Assisted Reproduction, Xinhua Hospital, School of Medicine, Shanghai Jiaotong University, Shanghai 200092, People’s Republic of China; ^2^ Department of Endocrinology, Shanghai Children’s Hospital, Shanghai Jiaotong University, Shanghai 200040, People’s Republic of China; ^3^ Department of Obstetrics and Gynecology, Ruijin Hospital, School of Medicine, Shanghai Jiaotong University, Shanghai 200023, People’s Republic of China; ^4^ Institutes of Biomedical Sciences of Shanghai Medical School, Fudan University, Shanghai 200032, People’s Republic of China

**Keywords:** bisphenol A, egg, HDAC7

## Abstract

Bisphenol A (BPA), a synthetic substance of endocrine disrupter, widely distributes in environment and can affect the health of ovarian follicles, thereby impacting the fertilization ability and pregnancy rate. However, the underlying mechanisms regarding how BPA disrupts the egg quality have not been fully revealed. In this study, we determine that BPA treated female mice display the decreasing HDAC7 expression in ovary and eggs compared to control. Moreover, the global levels of H3K9 and H4K16 acetylation abnormally increase after BPA treatment and recover partially upon HDAC7 compensation. Collectively, our study reveals that BPA deteriorates egg quality through HDAC7 suppression.

## INTRODUCTION

Bisphenol A (BPA) is a highly industrial volume chemical, and extensively used in the production of polycarbonate plastics and epoxy resins for containers, thermal receipt paper and dental sealants manufacture [1]. BPA, a representative endocrine disrupter (ED), mimics as estrogen-action and reduces follicle counts, fertilization and implantation rates, and sex steroid hormone production [2, 3]. Several government organizations recently give guidelines for the accepted lowest observed adverse effect level, but still overestimate the human exposure levels of BPA. Because BPA can potentially exert via lower doses and a nonmonotonic dose-response way [4].

Recent studies reveal that BPA produce an ovarian toxic effect through multiple pathways including free radicals generation, lipid peroxidation and apoptosis [5]. In addition, BPA can alter oviduct morphology to impair the development of conceptus and the movement from the oviduct to the uterus thus obstructing the implantation [6, 7]. Furthermore, BPA also affects uterine morphology and function through mechanisms that involve cell proliferation and receptivity [8]. However, most of these studies on eggs maturation were from clinical aspect. The mechanism that this endocrine disrupter finally impacts the overall cell fate is still less revealed.

Epigenetic modifications are heritable and reversible alterations responding to environmental stimulations that are not due to changes in genomic DNA sequence. The related studies have reported that DNA methylation decreased in genome-wide level [9], but increased in a portion of genes of some specific developmental pathway upon the different doses of BPA treatment [10]. Consistent with DNA methylation, BPA is supposed to be able to down-regulate H3K4me3 and up-regulate H3K27me3 respectively for gene transcriptional repression in ovary [11]. Moreover, BPA can also inhibit H3K9, H3K27 and H4K12 acetylation in testes [12]. However, the underlying mechanism bridge between BPA and histone deacetylases (HDACs) or histone acetyltransferases (HAT) remains poorly understood. This issue is important because the epigenetic associated enzymes, for instance, different classes of HDACs have different catalytic domains and participate in a variety of pathways and diseases [13]. HDAC5 was down-regulated in breast epithelial cells [14], and Sirt1 was not affected in ovarian cancer cells [15], and HDAC1 was up-regulated in cortical neuron [16] all after BPA exposure, which suggests that HDACs can be activated or suppressed through different pathways and they also have different preference for downstream targets selection. In this study, we focus on HDAC7, one of Class II HDACs, and investigate its molecular roles in eggs with BPA treatment, and try to figure out the epigenetic effect of BPA for egg maturation and vitality.

## RESULTS

### BPA can induce eggs granulation and reduce the fertilization rate

**Figure 1:**
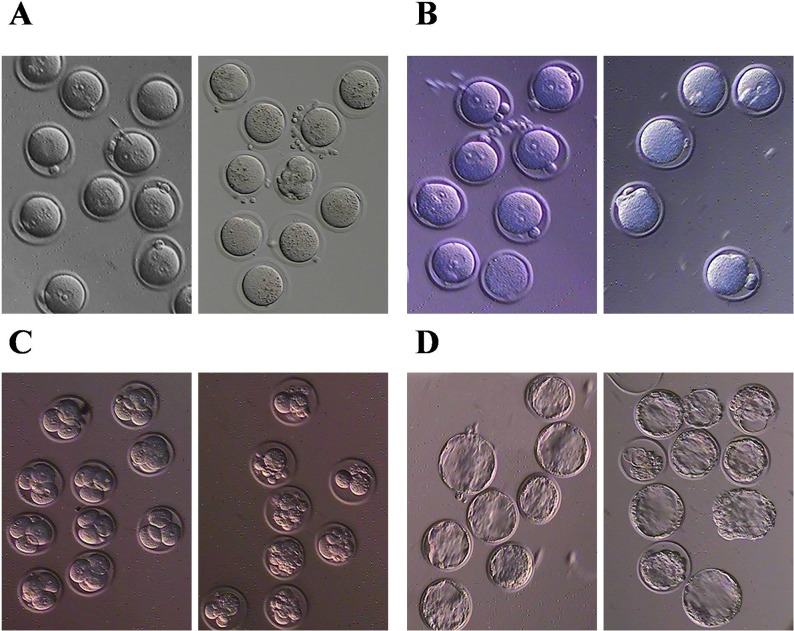
BPA can induce eggs granulation and reduce the fertilization rate **(A)** granulation of eggs, **(B)** fertilization, **(C)** early embryo development and **(D)** blastocyst development after BPA exposure. Left panel is normal control and right panel is BPA exposure.

To validate the toxic effect of BPA in our model, 79 eggs from the female mice treated with 50 μg/kgbw/day [17] BPA were harvested following a superovulation procedure, and were found that 58 eggs had dense granules in surface obviously compared to the 62 normal control. We considered the eggs with the granules filling in more than one third of surface as the granulation ones (χ^2^=37.045, *p<* 0.001) (Figure 1A). And the eggs with declining male pronucleus were also observed in BPA exposure compared to the control when they were received *in vitro* fertilization (IVF), which indicates the reduced ability of fertilization (χ^2^=5.877, *p=* 0.021) (Figure 1B). The unfertilized eggs were degraded in the next day even if some of eggs show the second polar bodies. Furthermore, BPA can also affect the development of zygotes at eight cell stage (χ^2^=17.620, *p<* 0.001). The zygotes treated with BPA display the cells with unequal size at eight cell stage in 3rd day (χ^2^=17.620, *p<* 0.001) (Figure 1C) and display the irregular shape of blastocyst with rough surface in 4th day (χ^2^=8.540, *p<* 0.001) (Figure 1D). Taken together, our study validated the toxic effect towards the status and fertilization rate of eggs.

### HDAC7 was down-regulated in egg and ovarian tissue after BPA exposure

**Figure 2:**
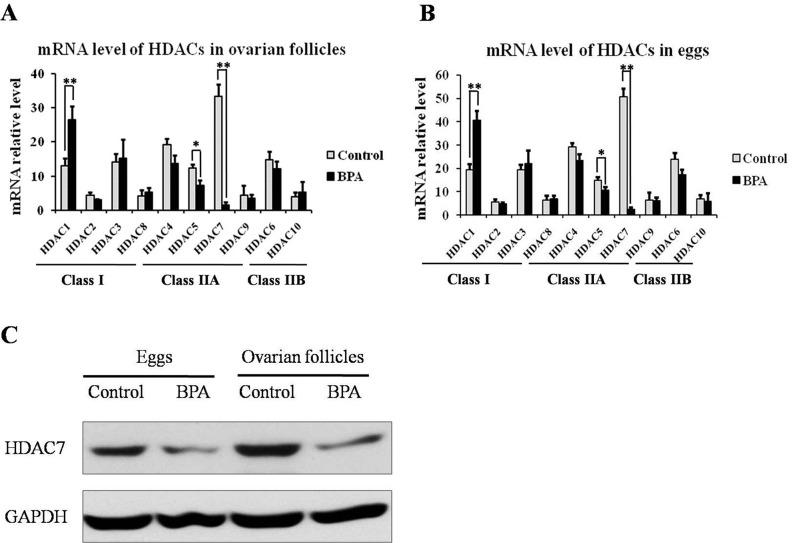
The expression level of HDACs in ovarian follicles and eggs upon BPA exposure The transcriptional level of HDACS in ovarian follicles **(A)** and eggs **(B)**. The protein level of HDAC7 in ovarian follicles and eggs **(C)**.

Then, we investigated HDACs mRNA level in eggs and ovarian tissues, and found that only HDAC1 were up-regulated in Class I HDACs, while HDAC5 and 7 was obviously down-regulated in Class II HDACS in ovarian follicles (Figure 2A) and eggs (Figure 2B) after BPA treatment compared to the control. In view of the most significant difference mRNA level of HDAC7 with BPA treatment or not, we further validated the protein level of HDAC7 in ovarian follicles and eggs (Figure 2C), and determined that HDAC7 in egg and ovarian follicles could be suppressed by BPA.

### HDAC7 was a specific target of BPA for epigenetic effect in egg development

**Figure 3:**
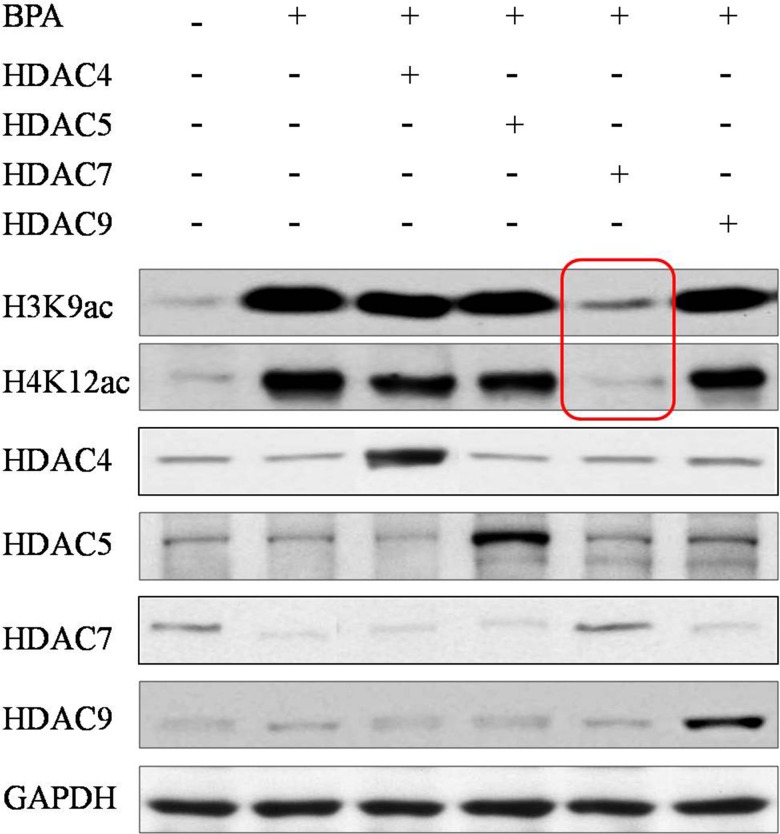
The global acetylation of H3K9 and H4K12 in eggs after HDACs compensation Red frames highlight that only HDAC7 compensation can recover the abnormal acetylation.

**Figure 4:**
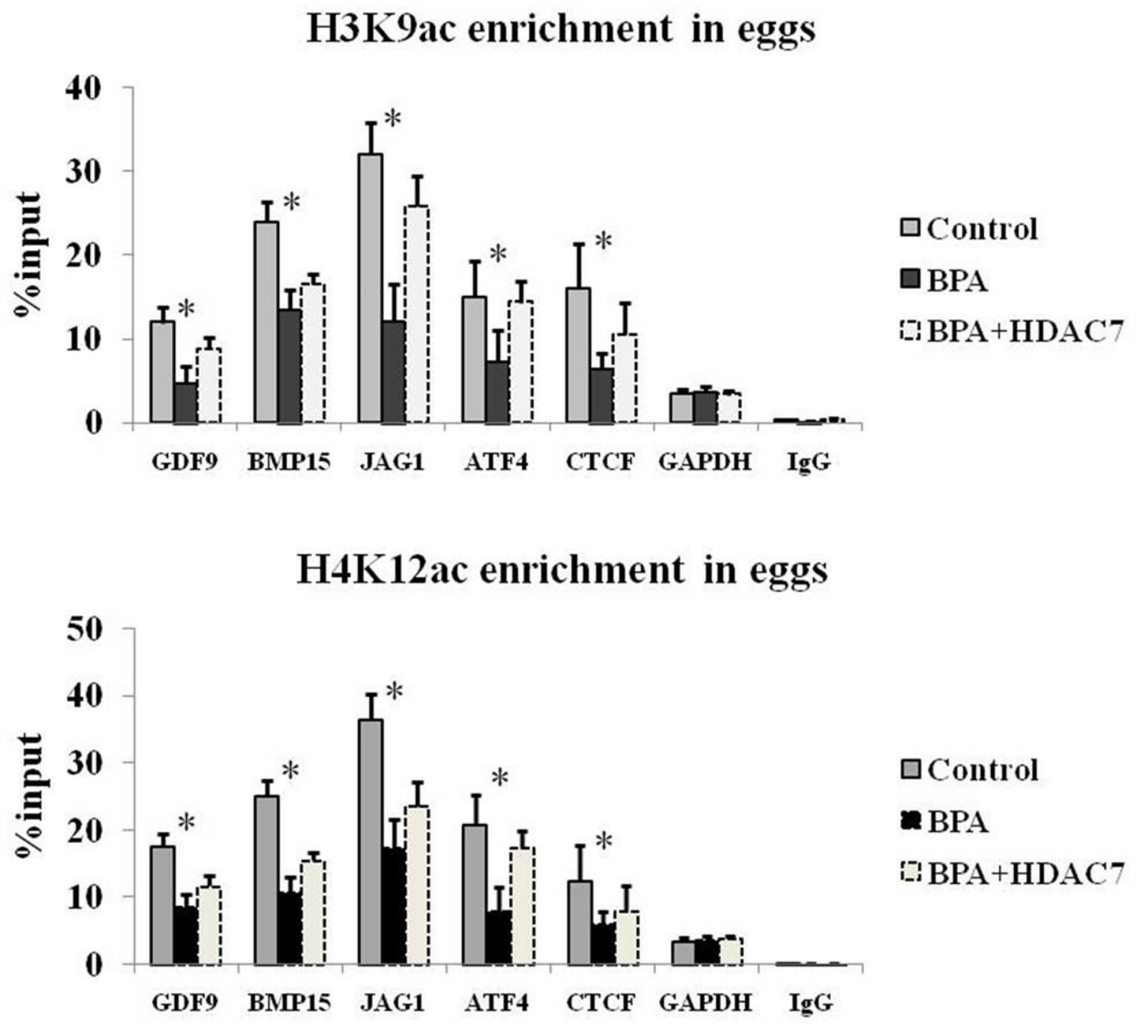
The H3K9ac and H4K12ac enrichment of relative genes in eggs

To investigate the effect of HDAC7 responding upon BPA in egg development, HDAC7 was over-expressed in eggs through microinjection and treated BPA simultaneously. We found that the global H3K9 and H4K12 acetylation were enhanced after BPA treatment compared to normal control (Figure 3). Interestingly, the recovery of these histone acetylation hallmarks were observed most obviously after the compensation of HDAC7 compared to the other HDAC5 and HDAC9 (Figure 3). In addition, we conducted H3K9ac and H4K12ac ChIP-qPCR assay to validate the promoter of genes associated with egg development, such as GDF9, BMP15, JAG1, ATF4 and CTCF. The presence of H3K9ac and H4K12ac loss and restoration at the promoter of these important genes upon HDAC7 and BPA treatment was observed (Figure 4). Taken together, we could determine that HDAC7 was the most sensitive target of BPA in Class II HDACs, and might play the important role in epigenetic maintenance during egg developmental process.

## DISCUSSION

BPA has been widely acknowledged to pose great risk to the multiple human diseases [18, 19], especially concerned with reproductive health and embryonic development [20]. And our results validate that BPA can affect oocyte maturation, fertilization rate and early embryonic development (Figure 1). The current studies state that BPA can bind to estrogen receptors and exert a weak estrogen-like and strong anti-androgen effect owing to the similar structure with estrogen [21, 22]. And hormone receptors are the key factors bridging the connection between environmental stimulation and response of epigenetic alteration [23, 24]. Therefore, there is one aspect of oocyte development which is generally stated to be impacted by BPA. This is HDACs.

Compared to Class I HDACs, Class II HDACs have more temporally and spatially restricted expression profiles and more specialized function as scaffolds for cellular processes. And Class II HDACs usually modulate epigenetic change for cell differentiation process via more flexible and alternative way, so that Class II HDACs silencing mostly cannot lead to individual lethality [25, 26]. Moreover, HDACs distribute in different cell type and have different targets for deacetylation in various biological events. And HDACs display different extent of response to HDACi or other chemicals, such as BPA (Figure 2A, 2B) even if they are under the same class. In our results, HDAC1 was enhanced by BPA, which is consistent with previous study [16], while HDAC5 [14] and HDAC7 was inversely down-regulated. Here, we determine that HDAC7 plays an important role in egg development, which was never reported previously.

Moreover, since the declining of HDAC7 expression is observed upon BPA exposure, we further compensate exogenous HDACs using microinjection and found that only HDAC7 can obviously compromise abnormal histone acetylation rather than other HDACs (Figure 3). Here, we determine that firstly BPA can suppress histone deacetylation mainly via HDAC7 silencing in eggs; secondly the roles of HDAC7 played in oocyte development cannot be replaced by other Class II HDACs. Previous study revealed that the expression profile of HDAC7 is different from other HDACs in oocyte development [27] and early embryogenesis [28], which indicates that HDAC7 has its specific targets that are contributed to the oocyte maturation and even embryonic development. Consistently, the enrichment of H3K9ac and H4K12ac at the promoter of GDF9, BMP15, JAG1, ATF4 and CTCF are obviously lost upon BPA treatment and retrieved after HDAC7 compensation (Figure 4). The H3K9ac and H4K12ac enrichment distributed in the promoter of these genes which play crucial roles in oocyte development are demonstrated to be the potential targets of HDAC7 in our study.

Taken together, our study reveals that HDAC7 is an important histone deacetylase for egg and early embryo development, and HDAC7 is also determined to be a potential target of BPA exposure, which can be considered as a targeted therapeutic strategy.

## MATERIALS AND METHODS

### Animal study

All the procedures were approved by the Institutional Animal Care and Use Committee of Shanghai, China. The outbred C57BL/6 mice were used in this study. Animals were fed with food and water ad libitum freely. Protocols were conducted to minimize pain and discomfort to the animals. 4-6 week old female mice with 16-20g weight were intraperitoneal injected with 10IU/0.1ml equinum serum and followed by 10IU/0.15ml human HCG after 48 hours, and sacrificed to harvest oocytes followed by 4 weeks. Meanwhile, the sperm were also harvested from male mice for IVF. Fertilization, embryos and blastocysts were observed at the second, third and fourth day respectively. Microinjection was carried out as described [29]. In brief, oocytes were culture with 1mg/ml hyaluronidase 2 hours for degranulation and washed by M2 medium, and microinjected with 2ng plasmid or PBS as control when the female pronucleus was observed fully and clearly. Plasmids of HDACs were cloned from mRNA of mouse tissue, and linked into PcDNA3.1. The primers used in this study were listed in Supplementary Table 1.

### Chromatin immunoprecipitation assay (ChIP assay)

Cells were collected and fixed with 1% formaldehyde-PBS for 15 min at room temperature for cross-linked chromatin preparation. The cross-linking reaction was stopped by the addition of 2 ml 125mM glycine buffer and trypsinized at 37°C. The precipitate was sonicated into 200-500 bp fragments. Antibodies of H3K9ac (Abcam, ab10812), H4K12ac (Abcam, ab46983) and IgG (Beyotime, Cat No. A7028) were incubated for IP reactions overnight. Protein A beads were used to pull down the antibody. DNA was extracted and purified for the real-time PCR assay.

### Real-time PCR assay

Total RNA was extracted from at least 100 eggs using Trizol methods. cDNA was produced from 1 mg of total RNA by iScript cDNA Synthesis kit (Bio-Rad, CA, USA) according to the manufacturer’s protocol.

Quantitative real-time PCR was performed using the SYBR Premix ExTaq Kit (Roche). The PCR protocol consisted of 35 cycles at 95°C for 30s, 40 cycles of 5s at 95°C, 30s at 60°C. The values were analyzed using the comparative CT method. The primers used in this study to detect the expression of HDACs were listed in Supplementary Table 1.

### Western blot

RIPA buffer with protein inhibitor cocktail (Roche, USA) was used for cell lysis. For immunoblotting, the transfer-ready membranes were blocked overnight in TBS (10mM Tris-HCl [pH 7.5], 150mM NaCl) containing 5% nonfat milk and 0.1% Tween-20 at 4°C, followed by incubation with appropriate primary antibodies. The secondary antibodies of horseradish peroxidase-conjugated anti-mouse, -rabbit, and -goat antibodies were used at a 1:5000 dilution.

### Statistical analysis

Chi-square test was used to analyze the morphology difference between BPA treated eggs and control. One way ANOVA was used to analyze Ct values of the real-time PCR data of the samples in different time points. Least significance difference (LSD) was used to further analyze between the two among the multiple samples. All analyses were processed by SPSS 20 software. *p* values less than 0.05 were regarded as statistically significant.

## SUPPLEMENTARY MATERIALS TABLE


