# Supplementary material for: Bisphenol A deteriorates egg quality through HDAC7 suppression
Source: Oncotarget. 2017 Sep 28;8(54):92359–65. doi: 10.18632/oncotarget.21308 (PMC5696187; doi:10.18632/oncotarget.21308)
Supplement: Supplementary file 1 [file oncotarget-08-92359-s001.pdf]

Bisphenol A deteriorates egg quality through HDAC7 suppression

SUPPLEMENTARY MATERIALS

Supplementary Table 1: All the primers used in this study were listed

Gene Symbol name	>Primer sequence	>Tm(°C)	
HDAC4	F: ATGAGCTCCCAAAGCCATC<br>R: CTACAGTGGTGGTTCCTCCTCC	52	For full length cloning
HDAC5	F: ATGCTGCTGGTGCCATGCTGCT<br>R: TTACCTGGGGCTGTGCTCTTGAGTGG	56	
HDAC7	F: ATGGACCTGCGGGTGGGC<br>R: CTAGAGGTTCATGGGTTCT	58	
HDAC9	F: ATGAGCTCGCCGGTGCAGC<br>R: TCACAAGGCTGGCTCCTC	55	
HDAC1	F: TGACTCACAATTTGCTG<br>R: CTGTATTCAGACATATTATCTGGGC	55	For qPCR assay
HDAC2	F: GGATCCATGGCGTACAGTCAAGGAGGC<br>R: GCGGCCGCTCAAGGGTTGCTGAGTTGTTC	60	
HDAC3	F: TTATGGTCTCTATAAGAAGATGAT<br>R: AGGCTCTTGGTGAAACCCTG	58	
HDAC4	F: CAGACACCCCTTGTCACAGGCCTGG<br>R: GCCTGTGCGTTCTGCGGCAGG	60	
HDAC5	F: GGCACCCAGCAAGCATCAGCAAGCA<br>R: ATGGGCATCACCACTGTCC	55	
HDAC6	F: CTAAATGACTTCCATTGCCTTT<br>R: AGGTGGGGCCAGAAGCGACCATGTTCA	55	
HDAC7	F: GCAGTCAGTCCACTCTGAAC<br>R: GCGAGCGGATGGCACTGAGG	60	
HDAC8	F: AATGATGCTGTCCTGGGAATATTACGATTG<br>R: GGAGAACTTGTGCAGGGACACAG	58	
HDAC9	F: GTAGCAGCTCTCCAGGGTCA<br>R: GCACTTCTGTTTGTCTTTGA	55	
HDAC10	F: CTTTGCCCATCTCACACAGCTG<br>R: GAAGGGGAGGTGTGGGGTCACC	58	
GAPDH	F: CGTCCCGTAGACAAAATGGT<br>R: TTGATGGCAACAATCTCCAC	58	
CDF9	F: AAAGTTTCCATTGCTGCTGCTC<br>R: TCTGATGTAACGGGCTCTTGG	60	For ChIP-qPCR assay
BMP15	F: tcccttgggcttgtgttggggcct<br>R: AAGTAGGGGCCTCAGCCAGAAG	60	
JAG1	F: TACTAGCGGTTTTACGGGCG<br>R: TCGAACAGGAGGAGCAGAGAGCGA	59	
ATF4	F: TCCCATTCTTCGGTGTAGCG<br>R: GTAACAAAGCAGTAGAGGCATTCG	59	
CTCF	F: GTGGGTCACAGCGGCAGATA<br>R: ACGATTGGGTAGTTCGGCATT	60	
